# Database covering the prayer movements which were not available previously

**DOI:** 10.1038/s41597-023-02196-x

**Published:** 2023-05-12

**Authors:** Senay Mihcin, Ahmet Mert Sahin, Mehmet Yilmaz, Alican Tuncay Alpkaya, Merve Tuna, Sevinc Akdeniz, Nuray Korkmaz Can, Aliye Tosun, Serap Sahin

**Affiliations:** 1 Department of Mechanical Engineering, Izmir Institute of Technology, Izmir, Turkey; 2 Department of Physiotherapy and Rehabilitation, Izmir Katip Celebi University, Izmir, Turkey; 3 Department of Mechanical Engineering, Istanbul- Cerrahpasa University, Istanbul, Turkey; 4 Department of Physiotherapy and Rehabilitation, Izmir Ataturk Training and Research Hospital, Izmir, Turkey; 5 Department of Computer Engineering, Izmir Institute of Technology, Izmir, Turkey

**Keywords:** Mechanical engineering, Preclinical research, Biomedical engineering

## Abstract

Lower body implants are designed according to the boundary conditions of gait data and tested against. However, due to diversity in cultural backgrounds, religious rituals might cause different ranges of motion and different loading patterns. Especially in the Eastern part of the world, diverse Activities of Daily Living (ADL) consist of salat, yoga rituals, and different style sitting postures. A database covering these diverse activities of the Eastern world is non-existent. This study focuses on data collection protocol and the creation of an online database of previously excluded ADL activities, targeting 200 healthy subjects via Qualisys and IMU motion capture systems, and force plates, from West and Middle East Asian populations with a special focus on the lower body joints. The current version of the database covers 50 volunteers for 13 different activities. The tasks are defined and listed in a table to create a database to search based on age, gender, BMI, type of activity, and motion capture system. The collected data is to be used for designing implants to allow these sorts of activities to be performed.

## Background & Summary

Quantification of motion has been of great interest to human beings. The very first studies were qualitative in nature and started by Edward Muybridge and his friends over photographic images of the locomotion of horses in the 1800s, leading scientists to the development of modern motion capture (MOCAP) tools^1^. Later, the use of reflective markers combined with LED (Light Emitting Diode) cameras has almost been a gold standard to collect accurate and precise data to describe motion of human body joints for the clinical assessment of patients in clinical settings^2,3^. The most common use of MOCAP systems is in gait laboratory applications. Gait analysis is to detect any deviations in patients motion pattern by comparing the collected data to healthy population data for diagnostic purposes or to assess injury level to plan an intervention and predict the outcome of an intervention^4,5^ in clinical settings. In addition to this, the output of gait data form the fundamentals of ISO standards to test the designed products against range of motion and loading conditions during a standard walking activity while testing orthopedic implants^6^. The kinematic and kinetic variables during a standard gait activity form the basis of implant design in the field of orthopedics. The designed products are tested against these boundary conditions to assess the wear volume and qualify to be implemented inside the patient’s body who requires artificial joint replacements.

Since human beings have a distinct pattern of walking, designing the products according to a standard walking pattern is believed to fulfill the basic and minimum requirement for mobility. The daily routine of a western human being mostly consists of walking, sitting, standing, climbing stairs, and cycling. However, in addition to these common activities, due to social, ethnic, and religious variations, there might be some other daily routines that require human beings to perform these activities frequently. These activities could be Salat activities^7,8^ which are performed five times daily, by the Islamic population of the world^9^, and yoga activities in Hinduism, also different sitting habits due to different layout and design of bathrooms in the West Asia, Central Asia, Middle East, and Far East part of the world, covering the crossed legged and squat type of sitting. Due to the cultural differences, the frequency of practicing these activities is higher when compared to the Western part of the world resulting in larger range of motions of joints and different loading patterns. Unfortunately, after implant surgeries, these patients are not supposed to follow their ritual practices, as the implants have not been designed for patients’ specific intended use^10^. Although there have been some studies investigating the performance of implants for these sorts of activities^8–14^, they are based on either hypothetical static posture in silico skeleton models or using an artificial skeleton using goniometers, inclinometers to use this information as an input to calculate stress and strain values in their models. Up to now, there is no existing open database covering the salat activities. However, if accurate data about a range of motion and force^15^ values could be calculated through a database using inverse kinematics, and kinetics, then the intended use-based products could be designed accordingly. For this reason, there is a need for building a database, which is collected from a population, performing these activities daily, using motion capture systems.

Although there are a few open access electronic databases that provide kinematic and/or kinetic data on human participants, including Asian Centered Database^16^, HuMoD Database^17^, Berkeley Multimodal Human Action Database^18^, CMU Graphics Lab Motion Capture Database^19^ and KIT Whole-Body Human Motion Database^20^ (Table 1) none of these data bases include salat activities. The Asian centered database consisted of 10 volunteers covering Chinese population only with a promise to extend the study to 500 people in total^16^. The HuMoD Database^17^ provides both raw and processed kinematic and kinetic data as well as electromyographically measurements for lower limbs tasks (e.g., walking, kicking ball, squatting) which were performed by one female and one male participant. The Berkeley Multimodal Human Action Database^18^ contains 11 actions performed by 12 participants. This database mainly comprises actions of high dynamics such as jumping jacks, throwing and hand clapping. The CMU Graphics Lab Motion Capture Database^19^ provides a wide range of motion capture data, including the interaction between human participants, sports activities (e.g., basketball, dance) and ADLs (e.g., sweeping floor, washing window). The KIT Whole-Body Human Motion Database^20^ captures the motions from 224 participants and 127 different objects such as cups, baskets and environmental elements such as staircases. These open access databases have recorded numerous motion capture data of healthy individuals. Nevertheless, none of these databases attempted to capture the motion of the West Asian or Middle Eastern population data and specific activities related to these geographies. Considering around a quarter of the world’s population is known to be Muslim, the number of people who might perform these activities five times a day, is estimated to be more than 2 billion, which is difficult to ignore^20^. Ethnicity is also known to influence anthropometrics^21–27^ for this reason it is crucial to collect the data from this part of world. In this study, we aim to include all these previously excluded activities by collecting data from the healthy population of West Asia and Middle East and share the data globally so that it is possible to improve the designs accordingly Table 1.

**Table 1:** Published databases from the literature using MOCAP systems covering daily life activities only.

Author-Year Database- Ref	No of subjects	MOCAP system	Population	Purpose
**Liang** ***et al****.***, 2020. Asian database**^16^	10 healthy 5 F, 5 M	Qualisys	Asian	Database for Asian population providing ADL MOCAP data was nonexistent, a database was created for benchmarking with non-healthy population for rehab purposes
**Wojtusch, & Stryk, 2015 HuMoD Database**^17^	2 healthy 1 F, 1 M	Qualisys	Germany	Kinematic and kinetic data as well as electromyographically measurements. over 8 lower limb tasks
**Ofli** ***et al****.***, 2013 Berkeley Multimodal Human Action Database (MHAD)**^18^	12 Healthy 5 F, 7 M	5 different system optical, multi-view stereo vision cameras, Microsoft Kinect cameras, wireless accelerometers, and microphones.	USA	To build multimodal database for action recognition purposes. 11 actions were demonstrated over 5 different multimodal MOCAP systems consisting of both lower and upper body tasks
**Torre** ***et al****.* **2009 Carnegie Mellon CMU Database**^25^,	26 healthy	Multimodal database; video, audio, (IMUs), RFID, Bodymedia, eWatch and motion capture	USA	Functional kitchen and cooking activities for activity recognition
**Mandery** ***et al****.***, 2015 KIT Whole-Body Human Motion Database**^20^	224 Healthy 37 F, 106 M	optical marker based Vicon MX MOCAP system, video recordings	USA	Wide range of motions from 38 subjects, includes environmental elements and objects, systematic structure and additional normalized motion representation
**Fukuchi 2018 Treadmill Database**^26^	42 healthy	Raptor-4; Mocap system, force plates, dual belt instrumented treadmills	Brazil	Walking data of both overground and on a treadmill at a range of gait speed to examine the influence of age, speed, environment on gait biomechanics for kinematic and kinetics
**Schreiber & Moissenet,2019 Full body marker different walking speed database**^27^	50 healthy 24 F,26 M	Qualisys, force plates, EMG	Luxembourg	To analyze the effect of walking speed on gait or conduct unusual analysis of gait thanks to the full body marker set
**Moore** ***et al****.***, 2015 Mechanical perturbation database**^21^	16 healthy 4 F, 12 M	Instrumented treadmill, Osprey camera Mocap system and accelerometers	USA	Three different walking speeds for validating or generating mathematical models that can simulate normal periodic gait and non-periodic, perturbed gaits

## Methods

### Participants

Participation of volunteers in medical research requires an ethics permission from an authorized committee. Therefore, to conduct *in vivo* study, ethics committee permission was granted from the Medical Faculty of Izmir Ataturk Research and Training Hospital. Informative documents for the volunteers, who will be participating in the study, were prepared for them to read and give their consent to participate in the study. Before the experiment, the Participant Information Sheet (P.I.S) and Participant Consent Forms (P.C.F of) were supplied to the volunteers to read and sign. The P.I.S informs the participant about the procedure, the data collection, the classified information, the exclusion criteria, the benefits, and the participant rights during the study. The P.C.F is obtained after the participant reads and understands the P.I.S. The Consent Form is vital for the experiment to start as it indicates the participant’s will to conduct the *in vivo* study. The participants’ questions are answered in detail in this stage of the study.

A total of 200 healthy participants (aged between 18 to 60) are to be recruited for this study. Exclusion criteria include: (1) having declared prior neurological conditions causing balance problems, surgeries, or medical conditions that need active medical intervention within the last six months, (2) declared visual problems that result in accidents or falls (3) having skin lesions or allergies that would hinder markers placement, (4) inability to participate normally in daily living tasks due to pain, (5) pregnancy, (6) having a BMI of more than 30 kg/m^2^. The participants were encouraged to wear appropriate attire for the data collection (e.g., shorts or exercise tights). Their clothing had no reflective material to avoid problems related to tracking markers. Up to now, 50 participants’ data from West Asia have been collected and statistical information is provided in Table 2.

**Table 2:** Basic demographics and body measurements of the participants.

**Age**	20–30	31–40	41–50	51–60
**Participant Number**	28	10	7	5
**Gender Ratio**	16 M 12 F	6 M 4 F	4 M 3 F	2 M 3 F
**Weight(kg) (Average** ± **SD)**	75.1 ± 13.7	67.6 ± 15.5	71.0 ± 17.8	72.4 ± 20.5
**Height(cm) (Average** ± **SD)**	175.4 ± 8.9	169.8 ± 9.3	172.0 ± 11.3	169.2 ± 15.8

### Experimental set-up and equipment

**Fig. 1:**
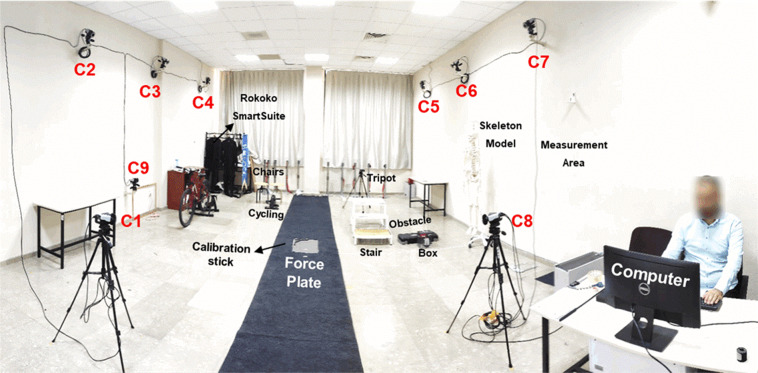
Laboratory settings demonstrating the layout and the used motion capture systems, and experiment tools.

All trials were conducted in the motion capture systems and biomechanics laboratory of Mechanical Engineering Department of Izmir Institute of Technology (IZTECH), Izmir, Turkey (Fig. 1). One of the motion capture systems used in this study is an optoelectronic marker-based system. Although this system is a gold standard used in gait analysis laboratories, it suffers from marker occlusion problem. For the subjects, where marker occlusion problem has occurred, due to their body type or because of their anthropometrics for the intended tasks, a wearable IMU based MOCAP system has been utilized. The detail of each system is provided below.

### Utilized motion capture systems

#### Marker based MOCAP system

**Fig. 2:**
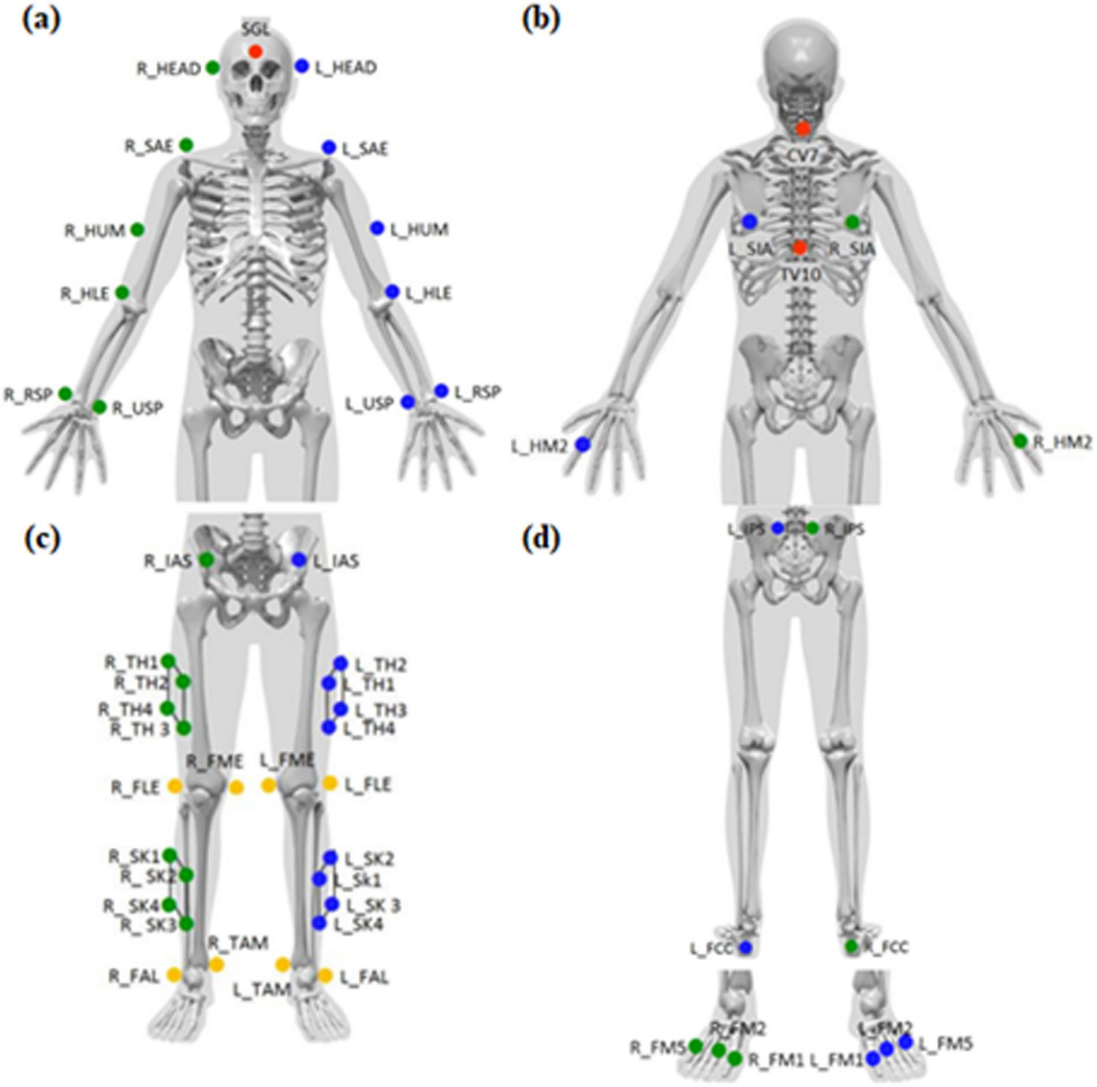
Marker placement set (**a**) Anterior View of CAST Upper Body Marker Set (**b**) Posterior View of CAST Upper Body Marker Set (**c**) Anterior View of CAST Lower Body Marker Set (**d**) Posterior View of CAST Lower Body Marker Set^22^.

Marker based optoelectronic system. The gold standard Qualisys system consists of 8 × Miqus M3 Cameras, 1 × Miqus Video Camera, 55 Super Spherical Markers for static measurements (47 for dynamic measurements) with Ø14 mm with Qualisys Track Manager (QTM) 3D Software and Visual 3D Gait Analysis Software. The data acquisition rate of the system is set to 100 frames per second (fps) for the experiment. The Qualisys system is integrated with a BERTEC portable force platform (Quincy, USA). QTM 3D Software, Visual 3D Software and a BERTEC force platform are used during data collection and related post processing tasks. The Qualisys uses reflective surface markers, which reflect the light back to the light emitting diode (LED) cameras for the image to be formed. The data sampling rate of the system is 100 fps. The 6 of the Miqus cameras are positioned on the walls and two of them were located on the tripods in the front as seen in the Fig. 1 to minimize the marker occlusion problem. The working range of the laboratory covers a volume of 8.4 m by 2.8 by 6.3 meter. The accuracy of the selected workspace is kept as 1 mm during the experiments. The workspace for the Qualisys MOCAP system is defined by calibration process. The calibration process starts by placing the L-frame to create the global coordinate systems of the laboratory. Then, the calibration wand is used to sweep the predetermined volume which contains the experimental setup with markers placed on it. To improve the accuracy of marker tracking, the threshold for accepting the standard deviation of the wand length is kept below 1 mm during the calibration process. Hence, the accuracy of the selected working volume is set to be 1 mm. Moreover, after the experiments, the average standard deviations of the marker positions are also calculated to confirm that they are below the threshold value of 1 mm. The preferred marker configuration is a CAST marker configuration which is shown in Fig. 2 and their anatomic location is explained in Supplementary Table 1.

### IMU Based Wearable MOCAP System

**Fig. 3:**
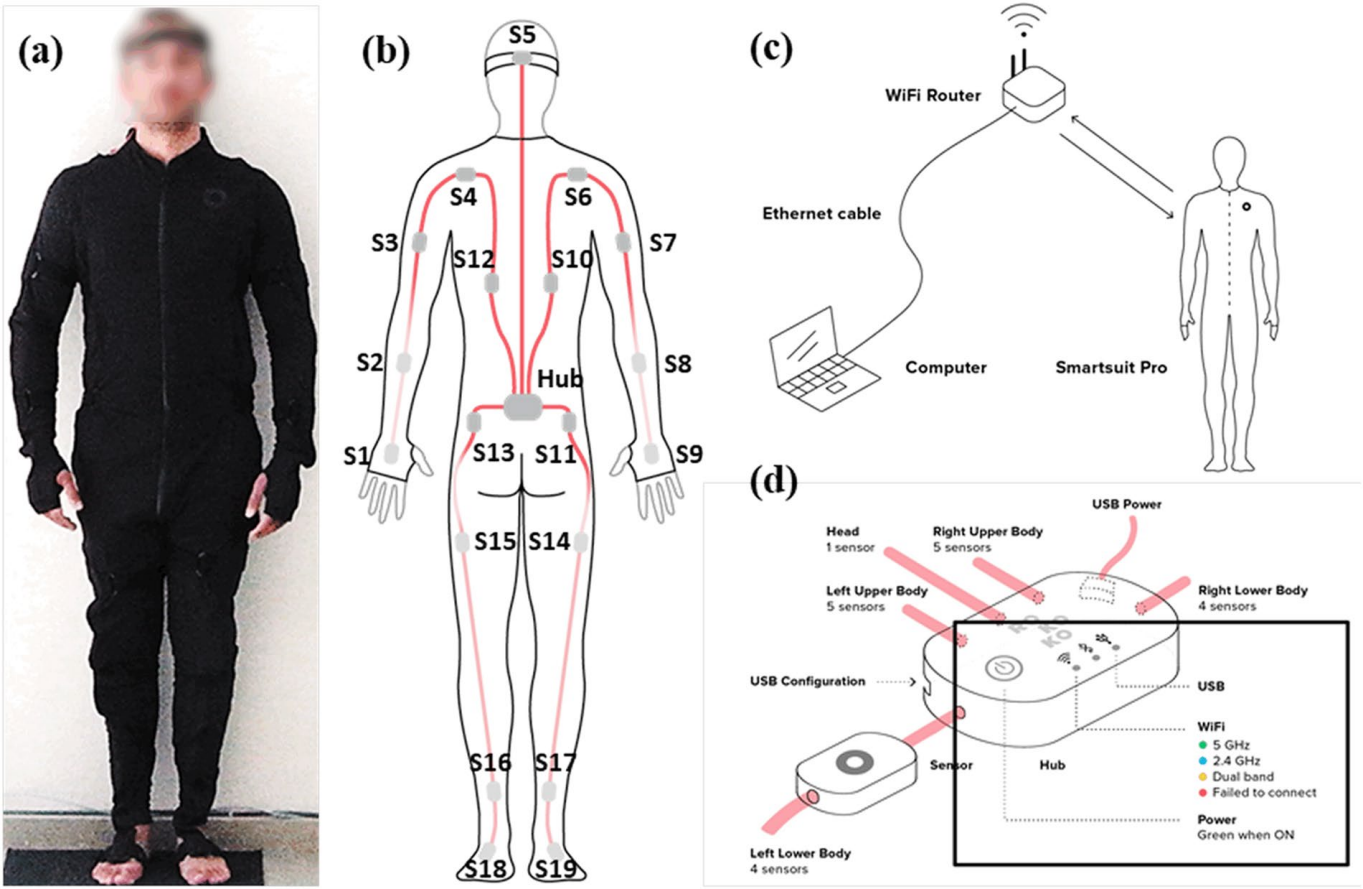
(**a**) Rokoko wearable motion capture device (**b**) the placement of the sensors in the suit (**c**) Communication Between Smartsuit and Computer^28^ (**d**) the hub collecting information from IMU sensors.

The wearable motion capture system Smartsuit Pro is a full body suit, which consists of 19 inertial measurement units (IMUs) Fig. 3. Each IMU has 9 degree-of-freedom (DoF), composed of 3 DoF accelerometer, 3 DoF magnetometer and 3 DoF gyroscope. The IMUs measure angular rate, acceleration, and magnetic field vectors in their local coordinate system and placed on the model with respect to the reference coordinate system which is defined in a calibration process known as sensor-to-segment calibration. Each IMU sensor’s orientation is obtained by a fusion algorithm Kalman Filter. The Extended Kalman Filter is a non-linear variant of the general Kalman Filter which is developed by SmartSuit Pro© (Rokoko, Denmark). The IMUs use quaternions, rotation matrices or Euler angles to define the segment’s orientation. To define the body segment orientations and positions, the acceleration readings are double integrated^23,24^. The Smartsuit collects the data and transfers it to The Smartsuit Studio Software to visualize the motions over a predefined model in real time. The data collection rate is 100 fps, which is the same for the Qualisys system. The Smartsuit has a unit which allows wireless communication between the computer and the IMUs to transfer data without disrupting motion^28^. The real time online monitoring of the motion through Smartsuit Pro Studio, allows identification of the drift visually, as the motion on the screen starts looking different from the original data. Moreover, the Smartsuit pro suit has a built-in warning system for drift or loss of data transfer in their built-in user interface, in case of such a problem the color of the sensors changes from green to yellow. If such a problem is observed, the experiment is halted, and the calibration process starts again to continue with the remaining tasks. Nevertheless, post processing is performed to observe any lost data during the process. It was observed that the completeness of the data transfer rate was in the range of 98 to 100 percent in the collected data files. The two percent missing data is filled by using interpolation techniques.

The raw data obtained by the IMU system are saved as BVH files. BVH files contain the position and orientation information of the left and right thigh, shin, foot, toe for the lower body for the recorded activities to analyze them in computational biomechanical modelling tools later for kinetic analysis. In case there is a need to directly read the position and orientation information of the recorded motions, the raw data could be saved as CSV file, a plain text file format used to store tabular data. However, in the database, we provided the output of the IMU based wearable MOCAP system in BVH file format to enable its use in biomechanical modelling programs.

### Study design

The study was designed in two stages. The first stage was providing proof of concept that SmartSuit Pro could provide comparable motion capture data to the marker based optoelectronic system. For this purpose, the data was collected from healthy volunteers using both systems simultaneously over single plane activities covering the full range of motion and the gait activities. The results of this study were published^15^ as evidence of proof of concept that the wearable MOCAP system could provide comparable results to the marker-based system. Due to the marker occlusion problem which could be observable during these previously excluded activities by some of the subjects, use of a system which is not affected by the line-of-sight problem, was required wherever possible.

In addition to the common daily life activities, which were included in the previous databases, salat postures, and different sitting styles are included in the database which are performed daily by West Asians, and Middle Eastern populations. The list of these activities is in Table 3 and their description is provided below. The data collection was performed initially using the marker-based system for all the activities, which does not suffer from marker occlusion problem. Most of the subjects were able to produce data without the marker occlusion problem, most frequently crossed legged sitting activity was recorded using the wearable suit. Following the static trial, subjects started performing the tasks, using the random number generation function using their sequencing order. The randomization procedure was planned to minimize any bias in performing the activities in the same order all the time.

**Table 3:** List of activities and their abbreviations used in this study.

Name of Activity	Short Form	Most Used MOCAP System
Qualisys static posture	Static	Qualisys
Gait	Gait	Qualisys/Smartsuit Pro
Obstacle Crossing	OBC	Qualisys/Smartsuit Pro
Stoop Lifting	STL	Qualisys/Smartsuit Pro
Squat Lifting	SQL	Qualisys/Smartsuit Pro
Asian Style Sitting	AST	Qualisys/Smartsuit Pro
Timed Up and Go	TUG	Qualisys/Smartsuit Pro
Chair Transfer	CHT	Qualisys/Smartsuit Pro
Cycling	CYC	Qualisys/Smartsuit Pro
Ruku’ and I’Tıdal	RAI	Qualisys/Smartsuit Pro
Ruku’ to Sujud	RTS	Qualisys/Smartsuit Pro
Sujud	SJD	Qualisys/Smartsuit Pro
Ascending Stairs	ACS	Qualisys/Smartsuit Pro
Descending Stairs	DCS	Qualisys/Smartsuit Pro
Crossed-Legged Sitting	CLS	Smartsuit Pro

### Procedure

**Fig. 4:**
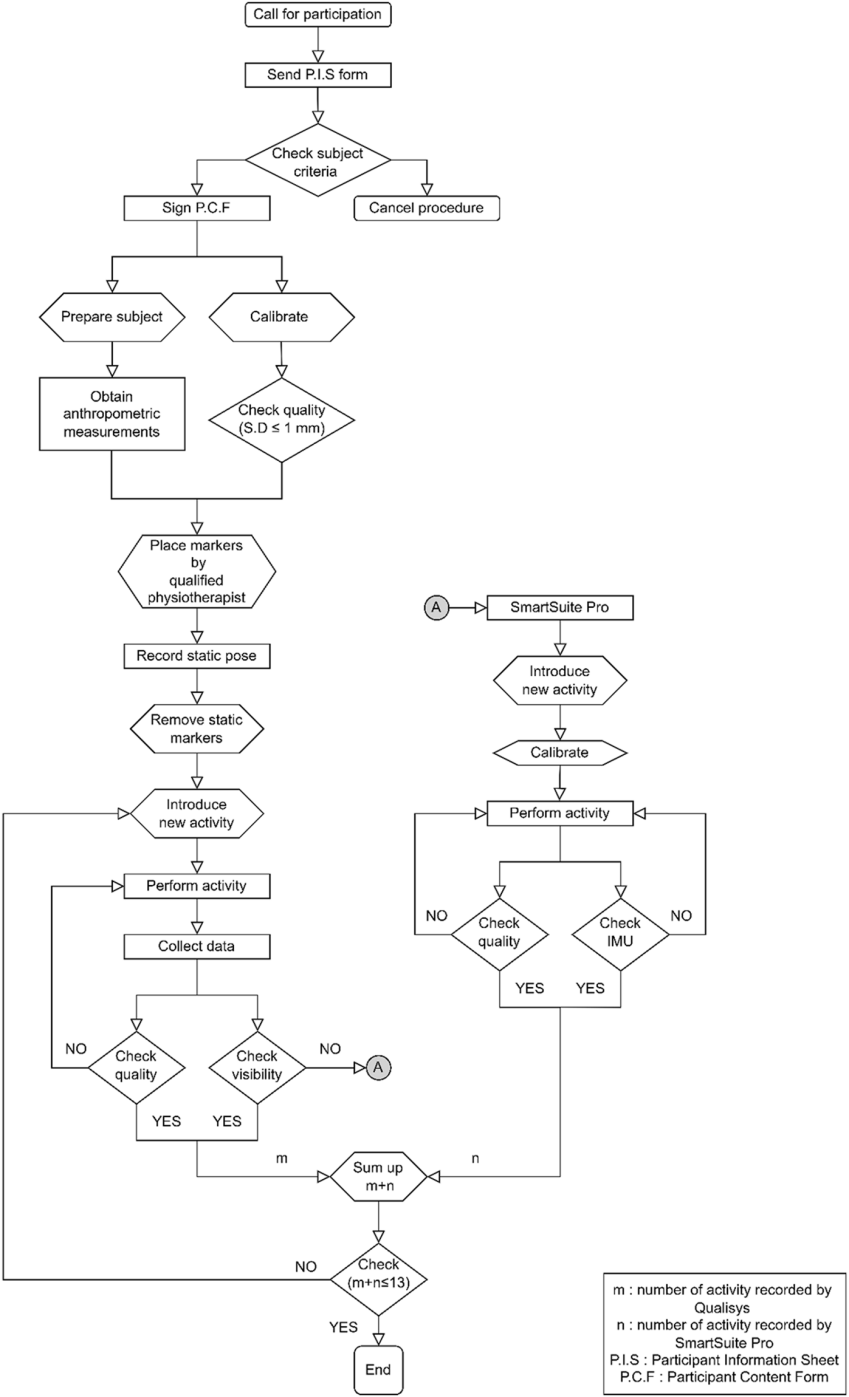
Flowchart demonstrating the decision points and action points during the experiment utilizing both MOCAP systems.

The algorithm demonstrating all the decision and action points for the experiment is provided in Fig. 4. The experiment starts with the marker-based system whenever line of sight problem is occurred, Then the list of activities which cannot be performed by the marker based MOCAP system is transferred to the wearable MOCAP system data collection procedure within the same session. All the activities are collected in the same session within two hours for ethical reasons.

All the experiment activities were performed with a designated dress code (e.g., shorts and tight singlet) that is suitable to CAST marker set. Any reflective accessories (e.g., earrings, clasp, necklace, and ring) that may disturb the data collection were removed. Anthropometric data of the participants were taken by a qualified physiotherapist. The reflective makers were placed onto the participant’s anatomical landmarks by the experienced physiotherapist according to the CAST marker set Fig. 2. For each activity a short naming format was utilized which is provided in Table 3 and used for file naming as well.

Before each activity, participants were informed about how they will perform the activities. Participants were asked to use their dominant leg to stand or step on force plate. When the participants were ready for the experiment, they started and ended each defined movement with sound notification. For each participant, static pose was taken for the purpose of building and scaling models in biomechanical simulation and analysis software. During static pose, the participant stands still for two seconds with the feet parallel to each other and the arms bent at ninety degrees.

### List of activities and their definitions in the database

#### Gait

**Fig. 5:**
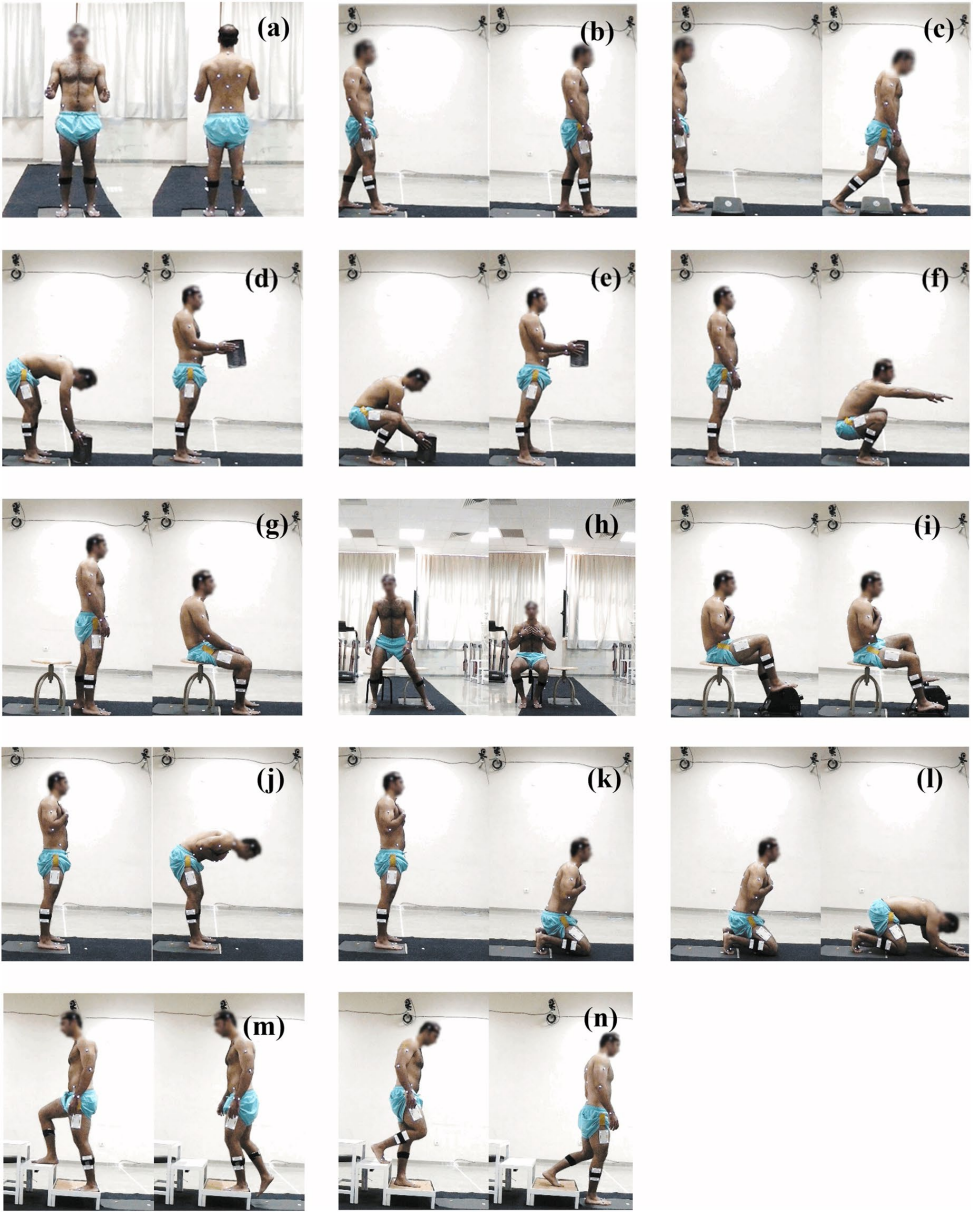
The qualysis figures of (**a**) QS, (**b**) gait, (**c**) OBC, (**d**)STL, (**e**)SQL, (**f**)ACS, (**g**) TUG, (**h**)CHT, (**i**) CYC, (**j**) RAI, (**k**) RTS, (**l**)SJD, (**m**) ACS, and (**n**) DCS.

**Fig. 6:**
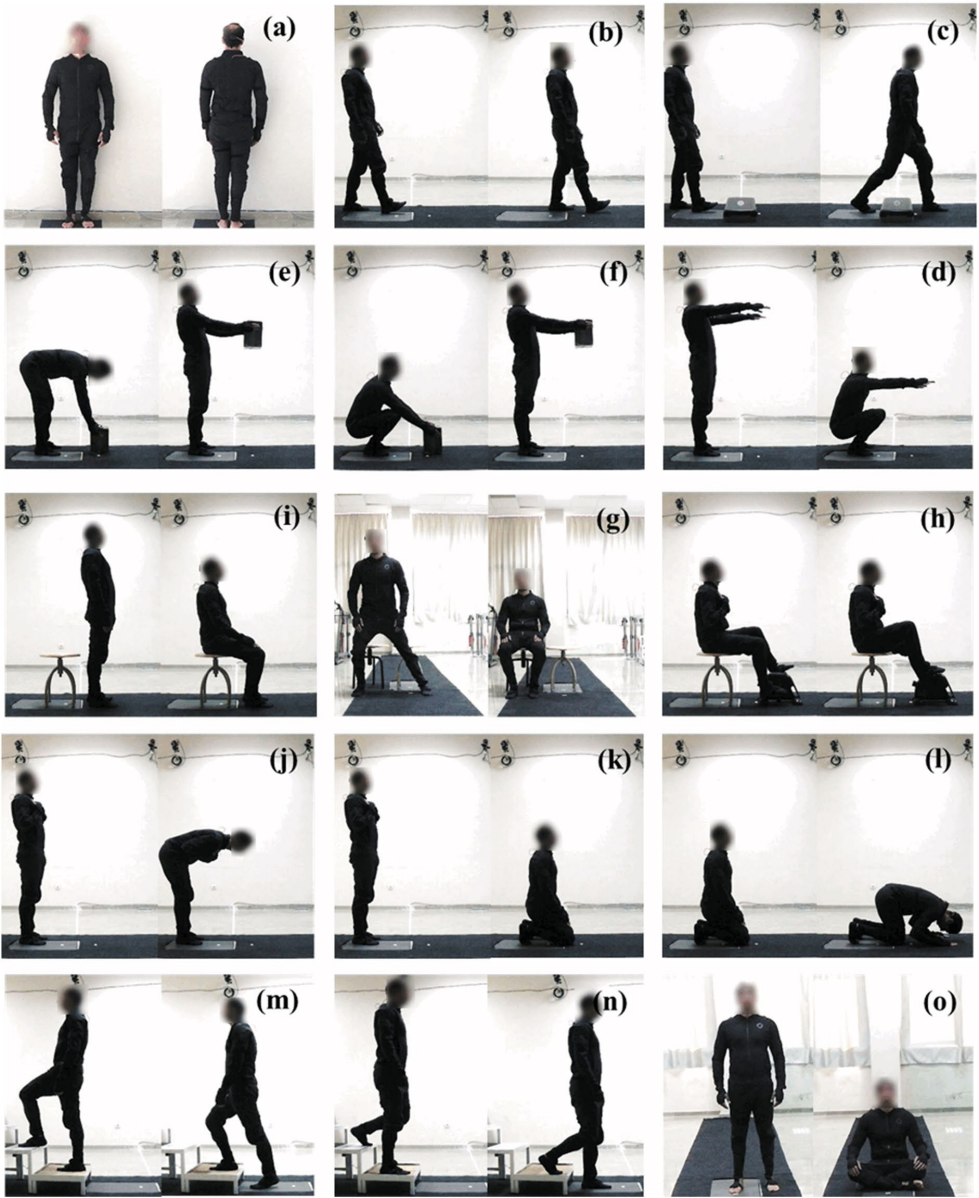
The smart suit figures of (**a**) QS, (**b**) gait, (**c**) OBC, (**d**)STL, (**e**)SQL, (**f**)ACS, (**g**) TUG, (**h**)CHT, (**i**) CYC, (**j**) RAI, (**k**) RTS, (**l**)SJD, (**m**) ACS, (**n**) DCS, and (**o**) CLS.

One of the most common worldwide DLA for all, is walking. Any disability or decrease in the capability of gait causes a loss in the level of comfort in a person’s life^29^. Therefore, maintaining a normal gait is a sign of having healthy lower limbs^30^. Clinically, kinematic, and kinetic analysis of gait have an importance to diagnose pathological conditions and evaluate the effectiveness of prosthesis and orthosis. The walking path was prepared to have a 5-m distance along the main axis of the laboratory with a width of 1 m. The participants were instructed to walk a 5-meter path at their own comfortable daily life walking speed on the path and to step on the force plate one foot at a time and with the other foot on the way back as shown in Fig. 5b. The gait cycle starts with the heel strike and ends with the heel strike of the same leg. Participants walked along the path until they got comfortable walking with the markers. The same activity was repeated with the wearable MOCAP system (Fig. 6b) for the subjects which suffered from marker occlusion problem.

#### Obstacle crossing (OBC)

Common situation encountered in daily life is the obstacles that appear on the way of movement path. In this activity, the movement of passing over obstacle without stepping on it was simulated using a commercial step board, the dimensions and the image are provided in the Table 4. The participant walked on the 1-meter-wide and 5-meter-long path and passed over the obstacle placed in front of the force plate. The cycle begins with the heel strike of foot on the force plate and ends with heel strike of the same leg after both legs pass the obstacle as shown in Fig. 5c. The same activity was repeated with the wearable MOCAP system (Fig. 6c) for the subjects which suffered from marker occlusion problem.

**Table 4:** List of accessory items used in the study and their dimensions, including the stair unit used for Ascending/Descending (ACS/DCS) stairs, chair for Timed Up and Go (TUG) and Chair Transfer (CHT), stepping board for Obstacle Crossing (OBC), box for Stoop Squat Lifting(STL/SQL) activities.

Name of Accessory items					
		Stair	Chair	Obstacle	Box
			
**Dimensions (cm)**	**∅**	—	36.5	—	—
	**w**	61.8	—	65.0	13.8
	**l**	119.2	—	29.0	51.3
	**h**	16.4	46.4	9.5	28.6

#### Stoop lifting (STL)

Lifting is a common activity used by the labor force or inactive people for the purpose of picking up an object from the ground. However, given the rate of repeated complaints and the damage it causes, lifting activity counts as a risk factor for low back pain (LBP)^31^. Understanding and analyzing the lifting activity has an importance on how to develop rehabilitation methods or exercises such as hamstring exercises^32^ to diminish the effects of LBP. The Stoop Lifting activity has two phases which are preparation and lifting phases. In the preparation phase, the participant holds a box which is made of carton and has a negligible weight (Table 4) including the dimensions, with their knees and elbows extended as shown in Fig. 5d. Then, in the lifting phase, the participant lifts the box by raising his/her trunk to erect position. One cycle starts from the fully flexed posture to the fully erect posture. The same activity was repeated with the wearable MOCAP system (Fig. 6d) for the subjects which suffered from marker occlusion problem.

#### Squat lifting (SQL)

Different techniques should be tried for lifting activity due to the hazards and damage it may cause. Since the squat lifting technique causes less disc compression and shear force, it is more preferred and recommended than the stoop lifting activity. Squat lifting activity has two phases which are preparation and lifting phases. In the preparation phase, the participant holds the box with elbow in extended and knees in flexed position (Fig. 5e). Then, in the lifting phase, participant lifts the box (as shown in Table 4 with the given dimensions) by raising his/her trunk to upright position. Initially the data was collected with the marker-based system. For the subjects who suffered from marker occlusion problem, the same activity was repeated with wearable MOCAP system (Fig. 6e).

#### Asian style sitting (AST)

The Asian style sitting is a common activity among Eastern populations. This type of sitting posture is generally used while using the bathroom. since public restrooms are still dominated by squat pans, which many find more hygienic due to the lack of thigh-and-toilet-seat contact. Since pelvis reaches below of the patella level, Asian style sitting resembles deep squat activity in terms of posture and movement (Fig. 5f). Asian style sitting is difficult to perform since deep flexion of the knee joint (>100°) causes overloading and negative effects on the knee stability and surrounding soft tissues^33^. The kinematic and kinetic analysis of Asians style sitting is crucial to investigate to understand safety levels to design implants according to this intended use. For this activity, the participants started with an erect posture. Then, the participants bent their knees until the pelvis reaches below the level of the knees. Volunteers were asked to keep their pelvis to the ground as close as possible. After the sitting phase, participants returned to their initial posture. Initially the data was collected with the marker-based system. For the subjects who suffered from marker occlusion problem, the same activity was repeated with wearable MOCAP system (Fig. 6f).

#### Timed Up and Go (TUG)

Timed up and go (TUG) activity is a comprehensive measure to evaluate physical functionality and stability. One of frequent use of timed up and go activity is to confirm the need for home health aide services for elderly people, especially those who show signs of osteoporosis and fragility^34^. Multiple movement transitions such as sitting, standing, walking, and turning makes TUG one of the most used activities in daily life. It is mostly assessed in pathological diagnoses and in orthotic and prosthetic studies.

The TUG activity also covers the ‘Sit to Stand’ activity. The researchers can post process TUG movement data to extract this information. To keep the experiment time limited to 2 hours for ethical reasons, TUG movement was performed for convenience. In this activity, the participants started walking towards the chair that was 3 meters away from them. After they sat on the chair, they were instructed to stand up and walk 3 meters away from the chair (Fig. 5g). During TUG activity, there is no pause in each phase of the task. Subjects perform each phase of the TUG movement continuously. The chair was located to force plate. Initially the data was collected with the marker-based system for the subjects who suffered from marker occlusion problem, the same activity was repeated with wearable MOCAP system (Fig. 6g).

#### Chair transfer (CHT)

Participants were instructed to start chair transfer activity on chair. The figure and the dimensions of the chair are provided in Table 4. Then, they were asked to get up from the chair and change to adjacent chair by performing sidestep (Fig. 5h). During the activity, participants were asked not to touch their bodies or get support while getting up from the chair. Initially the data was collected with the marker-based system. For the subjects who suffered from marker occlusion problem, the same activity was repeated with wearable MOCAP system (Fig. 6h).

#### Cycling (CYC)

The volunteer started this movement in the sitting posture and volunteers’ feet were positioned on the cycle pedal which is used as a home exercise device (commercially available minibike, Domyos 100). During the movement, the upper body remained stationary (Fig. 5i). A cycle for this movement was designated such that a marker on calcaneus makes one full rotation. This means that after one full rotation, the selected marker must return to its initial position. Initially the data was collected with the marker-based system. For the subjects who suffered from marker occlusion problem, the same activity was repeated with wearable MOCAP system (Fig. 6i).

#### Salat activities

In the 2010 census, the Muslim population was around 1.6 billion, and this number is expected to reach 2.2 billion by 2030^35^. As in every culture, there are specific activities and movements belonging to Muslim societies. The common activity among Muslim societies is Salat which is a praying activity. Since Salat is one of the conditions of being a Muslim, it is an activity that is repetitively performed five times a day. Examination of salat postures is important to understand its effect on the lower and upper extremities^36,37^ for designing implants according to this intended use. Salat is divided into three parts and investigated separately as Ruku’ and I’tidal, Ruku to Sujud, and Sujud. The data was collected from the subjects in this order, since salat requires this sequence.

##### Ruku’ and I’tidal (RAI)

One of the basic movements of salat is Ruku’ and I’tidal. The participants were asked to stand upright at the initial position of the activity. This upright position is called I’tidal. Ruku, on the other hand, is the act of placing the hands on the knees by bending the trunk forward while keeping the legs stationary (Fig. 5j). However, the participants were asked to place their hands on their chests not to cover or block the view of markers. For the subjects who still suffered from this problem, the wearable MOCAP system (Fig. 6j) was utilized.

##### Ruku to sujud (RTS)

Ruku to Sujud is one of the praying activities in Salat. The participants started this movement from the I’tidal posture (upright stance) of the prayer activity and sat down onto their knees before the prostration (Fig. 5k). Finally, the participants returned to their initial position (upright stance) and ended the activity. Initially the data was collected with the marker-based system for the subject who suffered from marker occlusion problem, the same activity was repeated with wearable MOCAP system (Fig. 6k).

##### Sujud (SJD)

In this activity, participants started in a prostrate sitting position (sitting on knees). Participants started the activity by moving their torsos in flexed position and placing their heads close to ground while keeping their hands onto ground (Fig. 5l). To return to the initial position, participants extended their torso and straightened their backs upright. Initially the data was collected with the marker-based system. For the subjects who suffered from marker occlusion problem, the same activity was repeated with wearable MOCAP system (Fig. 6l).

#### Stair climbing (STC)

Climbing stairs is frequently performed in daily life in order to go upstairs in the buildings or to go downstairs to underground transportation vehicles such as subways. It is important to examine stair climbing activity from a biomechanical point of view to aid in the improvement of stair gait and the optimal design of stairs used in public and work environments. Stair climbing was divided into two parts as stair ascending and stair descending. The staircase used in the activity had 3 steps and it was placed behind the force plate as seen in Fig. 6m,n.

#### Stair ascending (ACS)

Stair ascending consists of two phases, stance, and swing. The first phase is stance, and the second phase is swing. Participants started with initial contact to force plate with heel strike and ended with foot placement of the same leg that initially contacted onto second step. Each participant was instructed to ascend the stairs at a self-selected pace and place only one foot on each step (Fig. 5m). Initially the data was collected with the marker-based system. For the subjects who suffered from marker occlusion problem, the same activity was repeated with wearable MOCAP system (Fig. 6m).

#### Stair descending (DCS)

Participants started stair descending activity with double support of two legs on the second step. The activity ended with double support of two legs on ground after participants stepped on force plate embedded into ground. All participants were instructed to descend to stairs by placing only one foot on each step at a self-selected pace (Fig. 5n). For the subjects who suffered from marker occlusion problem, the same activity was repeated with wearable MOCAP system (Fig. 6n).

#### Crossed-legged sitting (CLS)

Crossed Leg sitting is one of the most common sitting postures in the Eastern cultures such as India, Japan, China, Middle East and Turkey, which have large populations^10^. Cross-legged movement was practiced on a flat surface. The participants were instructed to sit on the floor, cross-legged with the right leg at the bottom and the left leg on the top. Then hold this posture motionless for approximately 2 seconds. While ascending, participants were asked to change to the standing position at a comfortable pace for the subjects who suffered from marker occlusion problem, the same activity was repeated with wearable MOCAP system (Fig. 6o).

## Data Records

The data is stored in an open access database called APERTA which is provided by TUBITAK for researchers to keep their data safe and secure and share globally^38^. The filles in APERTA related to this study consist of c3d files and bhv files which are collected in this study. The researchers can access this open access database and download the full dataset. In addition to this, data is stored in the server of Biomechanics and Motion Capture Laboratory. (https://biomechlab.iyte.edu.tr/en/homepage/)^39^. It is possible to perform specific queries based on gender, age, BMI, and activity type, through this website different than the open access archive of APERTA.

Marker trajectories and synchronized force plate data were stored in a single c3d file. They are stored in c3d file format because it is supported in widely used biomechanics simulation and analysis software’s (Visual3D, AnyBody, OpenSim). Dynamic trials (in c3d format), static pose (in c3d format) and basic anthropometric data (in text format) of each participant were given in a separate folder. Labels, format, dimension, unit and description of each variable stored in the c3d files are given in Tables 5, 6.

**Table 5:** Analog data stored in c3d files (under Analog data).

Label	Format	Unit	Dim.	Description
Channel_01	Real	V	m × 1	Amplifier voltage outputs for channel 1
Channel_02	Real	V	m × 1	Amplifier voltage outputs for channel 2
Channel_03	Real	V	m × 1	Amplifier voltage outputs for channel 3
Channel_04	Real	V	m × 1	Amplifier voltage outputs for channel 4
Channel_05	Real	V	m × 1	Amplifier voltage outputs for channel 5
Channel_06	Real	V	m × 1	Amplifier voltage outputs for channel 6

(Refer to for https://www.bertec.com/s/Force-Plate-Manual_1_03_2021.pdf naming convention and formula used).

**Table 6:** Force plate data stored in c3d files.

Label	Format	Unit	Dim.	Description
Force	Real	N	m × 3	3D ground reaction force
Moment	Real	N.mm	m × 3	3D ground reaction moment
Position	Real	mm	m × 3	Center of pressure coordinates

The folders were renamed as ‘Sa####’, where #### presents the participant number for example, S0001 means that this folder contains the data of the first participant of our dataset. There are 16 activities (12 ADL and 4 Single Plane RoM Activity) from which the data were collected; the naming convention format is as ‘S####_G_A_B_T_P.’. Abbreviations for the naming convention are:

- S####: Participant number based the dataset, e.g., S0001,
- G: Gender, e.g., M(Male), F(Female),
- A: Age at the time of the experiment, e.g. A21
- B: Body Mass Index (BMI), e.g., B25
- T: Abbreviated task name, e.g., SJD (Sujud)
- P: Motion capture system, e.g., Q (Qualisys), S (Smartsuit)

An example of Qualisys data with an extension of c3d is as S0001_M_A21_B25_AST_Q.c3d

An example of a Smartsuit data with an extension of bvh and csv are as follows.

S0001_M_A21_B25_CLS_S.bvh S0001_M_A21_B25_CLS_S.csv

For the activity name, the naming convention of short form is used in CAPITAL LETTERS. (Table 3).

### Design of the database

The database was implemented as a part of the biomechanical laboratory website in Wordpress. The database requires functional operations on the website such as filtering system, login/logout, file upload page, list and delete files page. Since these functions could not be implemented via Wordpress, an additional web application was developed. The volunteers were recruited using the appointment system through the website and their anonymized data was uploaded to the same system upon completion. The desired main functions were created using Python and Django framework. For the backend side, Django was used for creating the model and Javascript for filtering functions. For the front-end, HTML, CSS, and JQuery were used.

### The django model-view-template

Django follows a Model-View-Template (MVT) architecture. The Model provides means for data access. The Template is a presentation layer which covers the User Interface part. The View is used to execute the business logic and interact with the model to carry the data and render the template Django uses Object-Relational Mapping (ORM), a technique that helps developers use databases without using any complex queries. Without Object-Relational-Mapping, developers would have to create the tables themselves and define the queries or procedures which requires translating plenty of SQL (Structured Querry Language) which is complex and hard to track.

### The design

The database was designed to keep the file names for filtering purposes and authorizing users to login and upload the files. The database system of Django called SQLite was used for this purpose. In the database table, the file names were stored under the media folder. This media folder is created in the Django project as default and stored all the files in this folder. One of the negative sides of keeping files in such a folder is when a file is damaged or lost, it is not possible to restore it. As a solution, all the original files are stored in another folder where none of the files can be modified. The users can filter the data based on the activity name, gender, age, and BMI values that they choose from the pull-down menu. The designed web site can be reached via: http://biomechlab.iyte.edu.tr/38. So that many other researchers will be able to make use of the excluded DLAs while building their own models for improving the quality of human life.

## Technical Validation

To understand the differences and limits of agreement between the two physiotherapists, gait kinematics results over the same participants were compared to provide evidence that the collected data is reliable. The gait activity was selected because the minimal detectable changes and similarity index have been suggested previously by many others in the literature. Therefore, the results of our experimenter dependency study could be compared with the existing literature. To assess intra-tester reliability of the two testers, the absolute mean differences in joint angles and the coefficient of determination (R^2^) deduced Linear Fit Methods were utilized.

Before the tests, the two testers were trained for placing the markers according to the CAST marker system (Fig. 2) through hands-on sessions to ensure that they are competent to conduct the experiments. The markers were placed by two different physiotherapists on three participants. The subjects were then asked to walk a 5-meter path at their own self-selected speeds. The sagittal plane angles of hip, knee and ankle joints at heel strike and toe-off were calculated in addition to the Range of Motion (RoM) for each trial. The mean angles and the standard deviations (SD) of the three subjects were calculated for each tester (Table 7). The mean differences and the standard deviation differences between the two physiotherapists were calculated (Table 7). The mean values of three subjects’ data were used to assess the waveform similarity (Table 8).

**Table 7:** Sagittal plane angles for tester A, B and their differences between Tester A and B during the heel strike, and the toe off events and the associated RoM values for the hip, knee and ankle joints.

		Heel Strike			Toe Off			RoM		
		Hip	Knee	Ankle	Hip	Knee	Ankle	Hip	Knee	Ankle
Tester A	Mean	29.12	0.34	71.77	4.65	−43.16	59.92	37.1	62.4	29.2
	Std. Dev	0.26	0.78	0.18	3.61	0.34	1.48	0.57	5.86	4.61
Tester B	Mean	29.43	0.84	72.38	8.79	−46.23	58.67	37.71	65.51	31.02
	Std. Dev	0.39	0.48	0.55	4.62	1.27	3.46	1.43	3.47	3.43
Tester A-B	Mean Differences	0.31	0.5	0.62	4.14	3.07	1.26	0.61	3.11	1.82
	Std. Dev Differences	0.13	0.3	0.37	1.00	0.93	1.98	0.86	2.38	1.18

**Table 8:** R^2^ obtained from Linear Fitting Method^40^.

	Hip	Knee	Ankle
R^2^	0.98	0.96	0.90

The calculated hip, knee and ankle mean sagittal plane joint angle differences are smaller than the reported minimal detectable changes of gait kinematics (<5°) (Table 8)^40^. The coefficient of determinations (R^2^) deduced The Linear Fit Method indicated waveforms were almost perfect fitted and all the R^2^ values were greater or equal to the acceptable value of the R^2^ reported Hip:0.98, Knee:0.96, Ankle:0.90) (Table 8)^41^. Moreover, Student’s t-Test was performed using two-tailed distribution with two-sample equal variance formula, the calculated p values were greater than >0.05 showing that the difference between the observers were insignificant (for hip 0.64, knee 0.1, ankle 0.47).

The use of IMU based studies gait analysis have been carried out for many years for healthy people and even for pathological gait identification^42^ to identify the kinematic gait parameters for people with gait problems. Moreover, authors provided the validation for the wearable MOCAP system, covering the flexion-extension, abduction-adduction, internal-external rotation range of motion and gait validation in a previously published work already^15^.

In this study, we intend to use the database for the design of lower body implants for the populations who perform the included activities. Although there are studies specific to upper body for spinal curvature^43^ and upper body joints^44^, this database provides data for the full body with a special focus on the lower body joints which could be considered as one of the limitations of the study. Moreover, the skin artefact problem which is an inherited confounding issue in MOCAP studies is valid for this study as well^2,15^. To investigate the effect of skin artefact problem, the same protocol could be repeated using a dynamic Magnetic Resonance Imaging (MRI) with MRI skin markers having reflective surfaces for their double use in Qualisys MOCAP system to investigate the correlation between the skin markers and the bone landmarks as a future work. However, this study stands out as the first database covering the salat activities to provide kinematic and kinetic information for biomechanical modelling. Next, the effect of skin artefact problem could be investigated in these activities.

## Supplementary information

Supplementary Table1

## Data Availability

The code written for the development of the database is available upon request from the authors, but it is not open to the external users through the website to protect the database. The desired main functions of the database were created in Python and Django framework. Django was used for creating the model for the backend and Javascript for filtering functions. For the front-end, HTML, CSS, and JQuery were used. The database is available through website https://biomechlab.iyte.edu.tr/en/homepage/38 and the public repository Database covering the previously excluded daily life activities | Aperta (ulakbim.gov.tr)^38,44^.
