# Author Correction: Database covering the prayer movements which were not available previously

**DOI:** 10.1038/s41597-025-06215-x

**Published:** 2025-11-04

**Authors:** Senay Mihcin, Ahmet Mert Sahin, Mehmet Yilmaz, Alican Tuncay Alpkaya, Merve Tuna, Sevinc Akdeniz, Nuray Korkmaz Can, Aliye Tosun, Serap Sahin

**Affiliations:** 1 Department of Mechanical Engineering, Izmir Institute of Technology, Izmir, Turkey; 2 Department of Physiotherapy and Rehabilitation, Izmir Katip Celebi University, Izmir, Turkey; 3 Department of Mechanical Engineering, Istanbul- Cerrahpasa University, Istanbul, Turkey; 4 Department of Physiotherapy and Rehabilitation, Izmir Ataturk Training and Research Hospital, Izmir, Turkey; 5 Department of Computer Engineering, Izmir Institute of Technology, Izmir, Turkey

Correction to: *Scientific Data* 10.1038/s41597-023-02196-x, published online 12 May 2023.

In this article the author’s name Nuray Korkmaz Can was incorrectly written as Nuray Can Korkmaz. The original article has been corrected.

